# Tula Hantavirus Infection in Immunocompromised Host, Czech Republic

**DOI:** 10.3201/eid1911.130421

**Published:** 2013-11

**Authors:** Hana Zelená, Jakub Mrázek, Tomáš Kuhn

**Affiliations:** Institute of Public Health, Ostrava, Czech Republic (H. Zelená, J. Mrázek);; University of Defence, Hradec Králové, Czech Republic (H. Zelená);; University Hospital, Ostrava (T. Kuhn)

**Keywords:** hantavirus, Tula virus, hantavirus pulmonary syndrome, hemorrhagic fever with renal syndrome, viruses, immunocompromised host

## Abstract

We report molecular evidence of Tula hantavirus as an etiologic agent of pulmonary-renal syndrome in an immunocompromised patient. Acute hantavirus infection was confirmed by using serologic and molecular methods. Sequencing revealed Tula virus genome RNA in the patient’s blood. This case shows that Tula virus can cause serious disease in humans.

Hantaviruses are enveloped RNA viruses carried by rodents and insectivore species. At least 5 hantavirus species are known to circulate in Europe: Dobrava-Belgrade virus, Puumala virus (PUUV), Seoul virus, Saarema virus, and Tula virus (TULV). The first 3 are well-characterized human pathogens; however, little is known about TULV human pathogenicity.

The species *Tulavirus* was first described by Plyusnin et al. (*1*) in voles (*Microtus arvalis* and *M. levis*) caught in Tula, Russia, in 1987. The presence of TULV was also documented in other vole species in several European countries including Germany, Switzerland, Slovenia, Czech Republic, Slovakia, Austria, Poland, and Serbia (*2*). In Central Europe, *M. arvalis* is the main reservoir of TULV. The TULV antigen was found in 10% of the population of common voles in southern Moravia in the Czech Republic (*3*). The pathogenic potential of Tula virus in humans is considered to be low.

The causative agents of hemorrhagic fever with renal syndrome in Central Europe are Dobrava-Belgrade virus and PUUV (*4*). These viruses seem to circulate in geographic areas that overlap with the areas where TULV circulates. Despite the massive population of common voles in the Czech Republic and a high prevalence of TULV in its rodent reservoir, human TULV infection has not been reported.

## The Patient

A 14-year-old boy from a rural region in the northeast part of the Czech Republic (Opava region) has received treatment for acute lymphoblastic leukemia since July 2011. Because of the biologic properties of the malignity, the boy was classified into the high-risk group of the treatment protocol. The intensive part of the treatment was finished in August 2012, and the patient has continued maintenance therapy since then.

During his first week of maintenance therapy, the patient experienced a respiratory infection with temperatures of ≈38°C, mild dyspnea, and a cough. These symptoms spontaneously disappeared. One week later, the patient had temperatures up to 38.5°C. He reported a headache, lack of appetite, and vomiting but no cough or respiratory distress. Upon the patient’s admission to the hospital, at the end of September 2012, his conditions deteriorated. He was febrile at 39.3°C and moderately dehydrated. Dyspnea with desaturation developed, so he was transferred to the intensive care unit to receive oxygenotherapy. The antileukemic maintenance therapy therefore had to be interrupted. The x-ray and high-resolution computed tomographic scan revealed severe bilateral bronchopneumonia with a major fluidothorax and bilateral dystelectasis. He was then given amoxicillin/clavulanate, amikacin, and antimycotic drugs. Oliguria also developed, with a minimum of 0.3 mL/kg/h, and it was managed by diuretic medication. Hemodialysis was not needed. He had transiently increased blood pressure followed by hypotension.

Laboratory results revealed eosinophilia in the patient’s differential leukocyte count at a maximum of 59.3% (reference range 0%–5%), anemia with a minimal value of hemoglobin of 60.0 g/L (reference range 135–175 g/L), thrombocytopenia at 12 × 10^9^/L (reference range 150–440 × 10^9^/L), and C-reactive protein 70 mg/L (reference range 0–10 mg/L). Elevated values were detected for serum urea measured at 8.40 mmol/L (reference range 1.8–6.4 mmol/L), creatinine at 103 µmol/L (reference range 27–88 μmol/L), and D-dimers at 3.53 μg/mL (reference range 0–0.5 μg/mL). Other coagulation parameters were not affected. Moreover, erythrocyturia and hyaline cylinders were observed in urine samples. The serum amylase and liver enzyme levels were within reference ranges. The relapse of acute lymphoblastic leukemia was excluded by the bone marrow examination. Because of the patient’s severe thrombocytopenia, thromboconcentrate was administered.

During the course of the patient’s hospitalization, his clinical condition, computed tomographic scan, and chest radiographic findings, and laboratory parameters improved. His renal failure gradually subsided with a transient polyuric phase. After 3 weeks of hospitalization, the patient resumed maintenance antileukemic therapy, and he was discharged from the hospital in good condition.

Serum samples taken on days 11, 12, 20, and 39 were tested for IgG and IgM antibodies to hantaviruses by using ELISA (Anti-Hanta Virus Pool 1 “Eurasia”; Euroimmun, Lübeck, Germany). The serum sample taken on day 12 was further tested for IgG and IgM antibodies by using Immunoblot (Anti-Hanta Profile 1; Euroimmun). ELISA results are considered positive when the index value (optical density divided by the cutoff value) is >1.1. Serology results suggested that the causative agent was a hantavirus antigenically closer to PUUV (Table 1).

**Table 1:** Results of hantavirus serologic testing, Ostrava, Czech Republic, October 2012 *

Virus	Serum samples obtained on day				Positivity range (IP)†
	11	12	20	39	
Hantavirus IgG ELISA	0.26	0.38	**2.69**	**2.60**	>1.1
Hantavirus IgM ELISA	**1.17**	**3.12**	**4.07**	**2.78**	>1.1
Puumala virus IgG Immunoblot	ND	Negative	ND	ND	ND
Puumala virus IgM Immunoblot	ND	**Positive**	ND	ND	ND
Dobrava virus IgG Immunoblot	ND	Negative	ND	ND	ND
Dobrava virus IgM Immunoblot	ND	Negative	ND	ND	ND
Hantaan virus IgG Immunoblot	ND	Negative	ND	ND	ND
Hantaan virus IgM Immunoblot	ND	Negative	ND	ND	ND

*Bold font indicates positive results. IP, positivity index; ND, not done.

†ELISA serology is considered positive when the index value (optical density divided by the cutoff value) is >1.1.

RNA was extracted from an EDTA plasma sample taken on day 11. Hantavirus RNA was detected by nested reverse transcription PCR performed with pan-hantaviral large (L) segment specific primers (*5*) (Table 2). Direct sequencing was performed with each separate nested primer and BigDye Terminator v1.1 Cycle Sequencing Kit (LifeTechnologies, Grand Island, NY, USA) on ABI 3130 platform.

**Table 2:** Primers used in the study, Ostrava, Czech Republic, October 2012

Primer	Step	Target segment	Sequence (5′→ 3′)	Reference
HAN-L-F1	1st PCR	Large	ATGTAYGTBAGTGCWGATGC	(*5*)
HAN-L-R1	1st PCR	Large	AACCADTCWGTYCCRTCATC	(*5*)
HAN-L-F2	2nd PCR, sequencing	Large	TGCWGATGCHACIAARTGGTC	(*5*)
HAN-L-R2	2nd PCR, sequencing	Large	GCRTCRTCWGARTGRTGDGCAA	(*5*)
S1	1st PCR	Small	GGMCAGACAGCAGAYTGG	(*6*)
S2	1st PCR	Small	AGCTCAGGATCCATRTCATC	(*6*)
MaS4F	2nd PCR, sequencing	Small	CATCACAGGSYTTGCACTTGCAAT	(*7*)
MaS5C	2nd PCR, sequencing	Small	TCCTGAGGCTGCAAGGTCAA	(*7*)

TULV RNA detection was confirmed by another PCR and sequencing experiment with small (S) segment Tula virus-specific primers previously published (*6*,*7*) for the first and second PCR step respectively (Table 2). The sequences were aligned to consensus sequence by using SeqScape software (Life Technologies) and compared with sequences available at BLAST database (http://blast.ncbi.nlm.nih.gov/Blast.cgi). Phylogenetic trees using neighbor-joining analysis with maximum composite likelihood method and bootstrap values were constructed by using MEGA5.2 software (http://www.megasoftware.net/).

**Figure 1:**
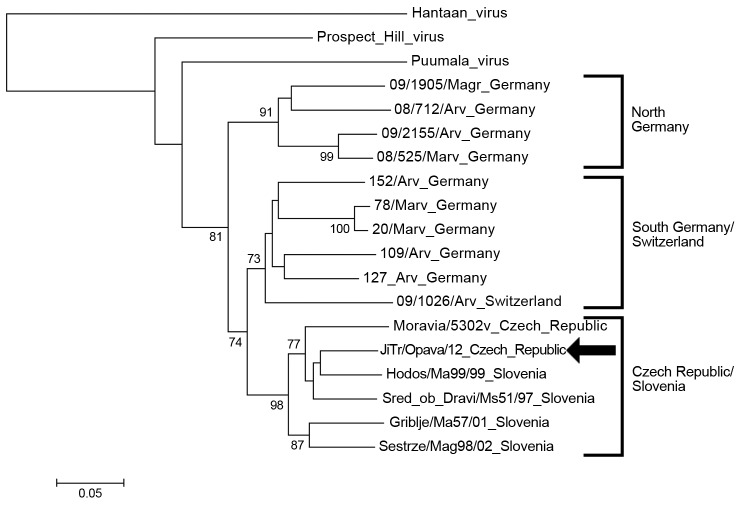
Phylogenetic tree (neighbor-joining analysis with maximum composite likelihood method) of Tula virus on the basis of large segment partial sequences (nt 2957–3337), Ostrava, Czech Republic, October 2012 GenBank accesion numbers: Haantaan virus (NC_005222), Puumala virus (Z66548), Prospect Hill virus (EF646763), 09/1905/Magr (HQ728460), 08/712/Arv (HQ728453), 09/2155/Arv (HQ728456), 08/525/Marv (HQ728461), 152/Arv (HQ728459), 78/Marv (HQ728464), 20/Marv (HQ728462), 109/Arv (HQ728457), 127/Arv (HQ728458), 09/1026/Arv (HQ728455), Moravia/5302v (AJ005637), JiTr/Opava /12 (KC522413), Hodos/Ma99/99 (FJ495101), Sred ob Dravi/Ms51/97 (FJ495102), Griblje/Ma57/01 (FJ495099), Sestrze/Mag98/02 (FJ495100). Bootstrap values ≥70%, calculated from 1,000 replicates, are shown at the tree branches. Arrow indicates strain isolated in this study. The tree is drawn to scale. The scale bar indicates an evolutionary distance of 0.05 substitutions per position in the sequence.

**Figure 2:**
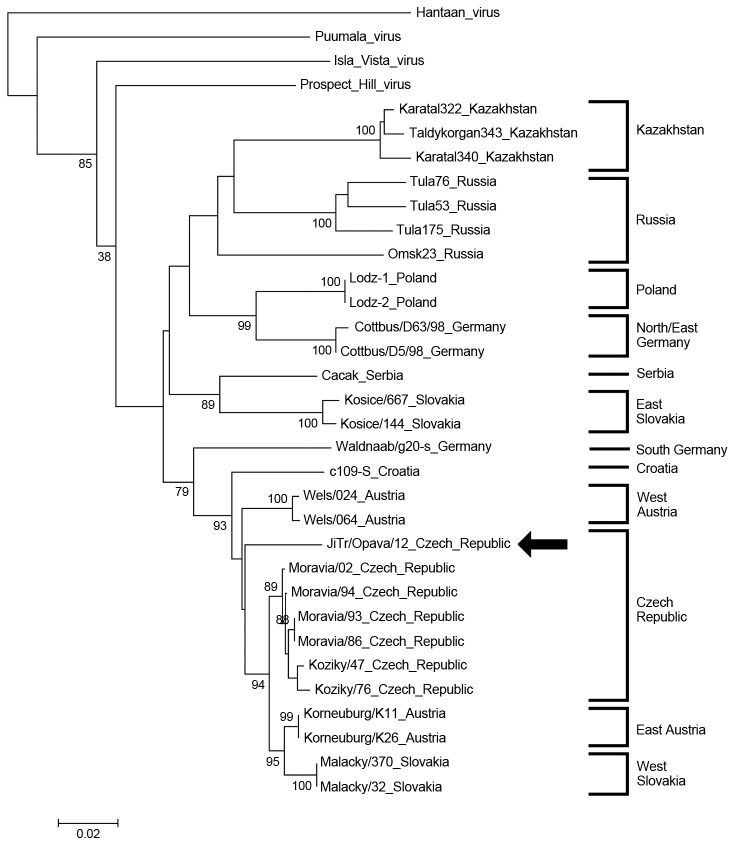
Phylogenetic tree (neighbor-joining analysis with maximum composite likelihood method) of Tula virus on the basis of small segment partial sequences (nt 428–758), Ostrava, Czech Republic, October 2012 GenBank accession numbers: Haantaan virus (NC_005218), Puumala virus (NC_005224), Prospect Hill virus (Z49098), Isla Vista virus (U19302), Karatal322 (AM945877), Taldykorgan343 (AM945879), Karatal340 (AM945878), Omsk23 (AF442621), Tula76 (Z30941), Tula53 (Z30942), Tula175 (Z30943), Lodz-1 (AF063892), Lodz-2 (AF063897), Cottbus/D63/98 (AF289821), Cottbus/D5/98 (AF289819), Cacak/Serbia (AF017659), Kosice/667 (Y13980), Kosice/144 (Y13979), Waldnaab/g20-s (AF164093), c109s (AF164094), Wels/O64 (U95309), Wels/O24 (U95302), JiTr/Opava /12 (KC494908), Moravia/02 (Z49915), Moravia/94 (Z48741), Moravia/86 (Z48573), Moravia/93 (Z48574), Koziky/47 (AJ223600), Koziky/76 (AJ223601), Korneuburg/K11 (U95305), Korneuburg/K26 (U95310), Malacky/370, Malacky/32. Bootstrap values ≥70%, calculated from 1,000 replicates, are shown at the tree branches. Arrow indicates strain isolated in this study. The tree is drawn to scale. The scale bar indicates an evolutionary distance of 0.02 substitutions per position in the sequence.

The EDTA plasma sample collected during the acute phase was positive for hantavirus RNA. Sequencing analysis of both L- and S-segments confirmed that the causative agent was TULV. The phylogenetic trees for partial L- and S-segments (Figures 1, 2) indicated that the identified Tula virus strain belongs to the lineage representing strains from middle Europe (Czech Republic, west Slovakia, Austria, and Slovenia). Partial L- and S-segment sequences of the TULV isolated RNA have been deposited in GenBank under accession numbers KC522413 and KC494908, respectively.

## Conclusions

Although the presence of TULV in the common vole population in the Czech Republic has been documented, no evidence of its pathogenicity in humans has been shown. Specific antibodies against TULV have been identified in a healthy blood donor in the Czech Republic (*8*) and in German forestry workers (*9*), suggesting that TULV can be transmitted to humans. A case of a serologically detected symptomatic TULV infection that followed a rodent bite has been reported in Switzerland (*10*). However, because of the late occurrence of specific antibodies and because the symptoms were atypical for hantavirus infection, the evidence for the Tula virus as an etiologic agent in this case is questionable (*11*). Renal and pulmonary syndrome with biphasic course associated with TULV was documented in northern Germany. The diagnosis was made on the basis of the highest neutralizing titer against TULV and detection of TULV RNA in common voles in the region where the patient lived (*12*).

We provide the molecular evidence of human symptomatic TULV infection. The clinical symptoms included both renal and pulmonary involvement with dominating respiratory failure corresponding to the hantavirus pulmonary syndrome. The course of the disease was severe, and the delayed occurrence of TULV IgG was most likely caused by the patient’s immunodeficiency. The laboratory findings were typical for hantavirus infection, with strongly decreased platelet count but only moderately elevated serum creatinine and urea.

Furthermore, during the acute stage, viral RNA was detected in the patient’s serum, which strongly suggests that TULV is a causative agent of the critical stage. This case illustrates that TULV can cause life-threatening disease in an immunocompromised patient, although under normal circumstances it is a nonpathogenic virus (*8*).
